# Direct synthesis of hydrogen peroxide from plasma-water interactions

**DOI:** 10.1038/srep38454

**Published:** 2016-12-05

**Authors:** Jiandi Liu, Bangbang He, Qiang Chen, Junshuai Li, Qing Xiong, Guanghui Yue, Xianhui Zhang, Size Yang, Hai Liu, Qing Huo Liu

**Affiliations:** 1 Institute of Electromagnetics and Acoustics, Department of Materials Science and Engineering, College of Materials, Xiamen University, Xiamen 361005, China; 2 Fujian Provincial Key Laboratory of Plasma and Magnetic Resonance, Institute of Electromagnetics and Acoustics, Department of Electronic Science, Xiamen University, Xiamen 361005, China; 3 Key Laboratory of Special Function Materials & Structure Design of the Ministry of Education, Key Laboratory for Magnetism & Magnetic Materials of the Ministry of Education, and School of Physical Science & Technology, Lanzhou University, 222 South Tianshui Road, Lanzhou 730000, China; 4 State Key Laboratory of Power Transmission Equipment & System Security and New Technology, Chongqing University, Chongqing 400044, China; 5 Department of Materials Science and Engineering, College of materials, Xiamen University, Xiamen 361005, China; 6 Department of Electrical and Computer Engineering, Duke University, Durham, NC 27708, USA

## Abstract

Hydrogen peroxide (H_2_O_2_) is usually considered to be an important reagent in green chemistry since water is the only by-product in H_2_O_2_ involved oxidation reactions. Early studies show that direct synthesis of H_2_O_2_ by plasma-water interactions is possible, while the factors affecting the H_2_O_2_ production in this method remain unclear. Herein, we present a study on the H_2_O_2_ synthesis by atmospheric pressure plasma-water interactions. The results indicate that the most important factors for the H_2_O_2_ production are the processes taking place at the plasma-water interface, including sputtering, electric field induced hydrated ion emission, and evaporation. The H_2_O_2_ production rate reaches ~1200 μmol/h when the liquid cathode is purified water or an aqueous solution of NaCl with an initial conductivity of 10500 μS cm^−1^.

Hydrogen peroxide (H_2_O_2_) has found many applications in modern industry including acting as a strong oxidizer, a bleaching agent, disinfectant, and the propellant in rocketry^1^,^2^ etc. due to the specific property of its oxygen-oxygen single bond. At present, the industrial production of H_2_O_2_ is dominated by the anthraquinone process which needs multistep processes and consumes much energy^3^. Therefore, a direct synthesis of H_2_O_2_ is required to avoid the disadvantages of anthraquinone process. Obviously, H_2_O_2_ synthesis directly from its constituent elements, i.e., H_2_ and O_2_, is a simple idea^4^,^5^,^6^, but the mixture of H_2_ and O_2_ is explosive. Some alternative direct methods do not use H_2_ and O_2_ which avoid the explosive problem and enable the *in-situ* continuous synthesis of H_2_O_2_, such as electrochemical synthesis from O_2_ and water^7^,^8^,^9^,^10^,^11^, photocatalytic TiO_2_ in aqueous solutions^12^,^13^,^14^,^15^, and even sunlight-driven production from water and O_2_ although the production rate is relatively low (0.625 μmol/h)^16^.

It has been proven that H_2_O_2_ can be formed by the reaction of H_2_ + O_2_ → H_2_O_2_ in plasma containing H_2_ and O_2_ gases^17^,^18^,^19^. When plasma is in contact with water, the plasma-water interactions can entail many direct reactions at the plasma-water interface and indirect cascade reactions in the bulk water^20^,^21^,^22^,^23^,^24^,^25^. One important species at the plasma-water interface is hydroxyl radical (OH) produced by plasma-induced water reactions with electrons and ions. The exact OH formation pathways by plasma-water interactions are very complicated, and one can refer to a review paper^26^ for details. As the building blocks, the generated OH radicals combination contributes to the main process of H_2_O_2_ formation in plasma-water interactions. In addition, other less probable pathways such as OH + H_2_O^*^ → H_2_O_2_ + H^27^,^28^ are also possible. In fact, there exist many reports on the H_2_O_2_ formation by discharge plasma operated over and inside aqueous solutions^27^,^28^,^29^,^30^,^31^,^32^,^33^,^34^,^35^. However, the determining factors which influence the H_2_O_2_ production from the plasma-water interactions remain unclear. To optimize the H_2_O_2_ production from plasma-water interactions, a better understanding of the H_2_O_2_ production process is desired. Herein, we present an insight into the understanding of H_2_O_2_ production by the plasma-water interactions. The results indicate that the sputtering, the electric field induced ion emission, and the evaporation at the water surface strongly influences the H_2_O_2_ production.

## Results

### Experimental approach and the H_2_O_2_ production

**Figure 1:**
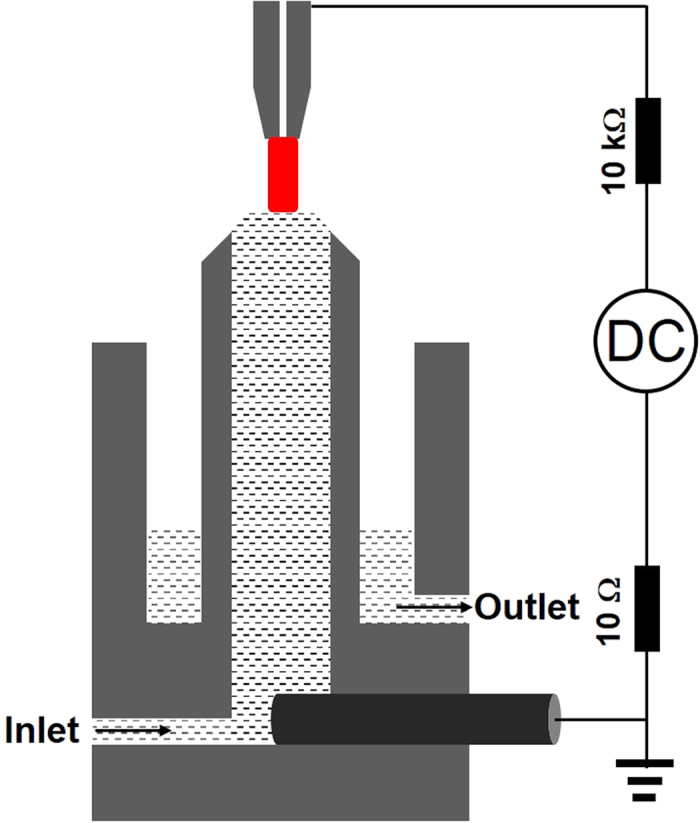
Schematic diagram of the device for the H_2_O_2_ production.

H_2_O_2_ was produced by the device schematically shown in Fig. 1 [see Supplementary Information (SI) for details]. A direct current Ar atmospheric pressure plasma was generated between a tungsten steel tube (1.02 mm and 6.35 mm in inner and outside diameters, respectively) and a liquid surface. The liquid acts as cathode or anode (positive or negative voltage applied to the tungsten steel electrode), and NaCl was used to adjust the initial conductivity of the liquid. The Ar flow rate, the discharge current, and the gap between the tungsten steel tube and the liquid surface were set to be 20 sccm, 30 mA, and 3 mm, respectively. To refresh the surface liquid, the total 400 ml liquid was circuited by a peristaltic pump at a flow rate of 200 ml/min. During the plasma-solution interactions, the H_2_O_2_ yield was measured at a given interval of 10 min, and the temperature, pH value, and the conductivity of the solution were also investigated.

**Figure 2:**
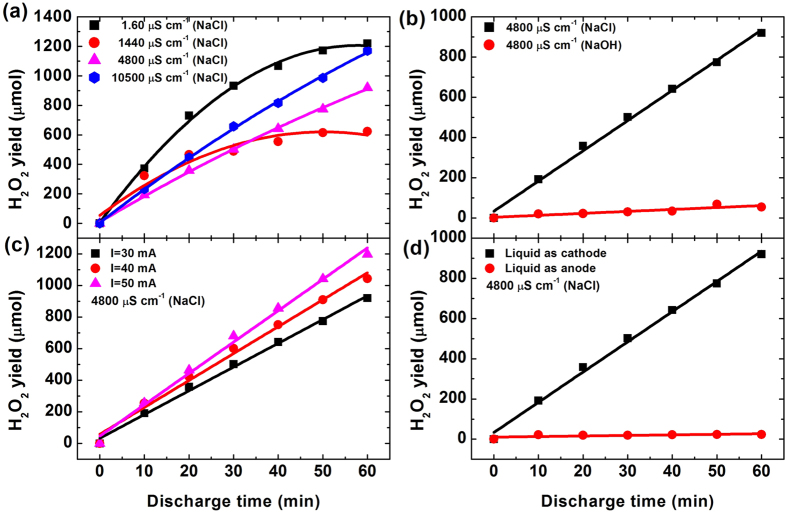
H_2_O_2_ yields at different experimental conditions. Plasma-liquid interactions were performed with liquids of **(a)** initial conductivities of 1.60, 1440, 4800 and 10500 μS cm^−1^, **(b)** NaCl and NaOH with the same initial conductivity of 4800 μS cm^−1^, **(c)** NaCl with the initial conductivity of 4800 μS cm^−1^ at discharge currents of 30 mA, 40 mA and 50 mA, and **(d)** NaCl with an initial conductivity of 4800 μS cm^−1^ (The liquid acts as cathode or anode).

Figure 2 presents the H_2_O_2_ yields at different experimental condit ions. For liquids with low initial conductivities (1.60 μS cm^−1^ and 1440 μS cm^−1^), the H_2_O_2_ production rate (the slope of the yield curve) decreases with increasing time, while they are constants for the liquids with high initial conductivities (4800 μS cm^−1^ and 10500 μS cm^−1^) [Fig. 1(a)]. Even with an initial conductivity of 4800 μS cm^−1^, the H_2_O_2_ yield for NaOH solution is almost zero after 60 min plasma treatment [Fig. 1(b)]. As the discharge current increases, the H_2_O_2_ production rate is enhanced [Fig. 1(c)]. When the liquid acts as anode (negative voltage applied to the tungsten steel electrode), there is almost no H_2_O_2_ production [Fig. 1(d)]. These results guide us to a question: What is the underlying mechanism for these differences? To answer this question, we must carefully analyse the processes taking place at the plasma-liquid interfaces from which the H_2_O_2_ is formed during the plasma-liquid interactions.

### Water molecule transfer processes at the plasma-liquid interface

When the liquid acts as cathode, a cathode voltage fall (*V*_*C*_) will be built between the plasma and the liquid surface due to the space charge accumulation^36^,^37^,^38^,^39^,^40^. The *V*_*C*_ will be located at a limited distance called cathode sheath, and for an atmospheric pressure discharge plasma, the thickness of the cathode sheath is smaller than 100 μm when the discharge current is more than 5 mA^36^. In our case, the discharge current is 30 mA, it is reasonable to take the sheath thickness as 100 μm. Table 1 presents the *V*_*C*_ for liquids with different initial conductivities (see SI for the details of the *V*_*C*_ estimation). The following analysis will demonstrate that this cathode region near the liquid surface is very important to the water molecules transfer at the plasma-liquid interface.

**Figure 3:**
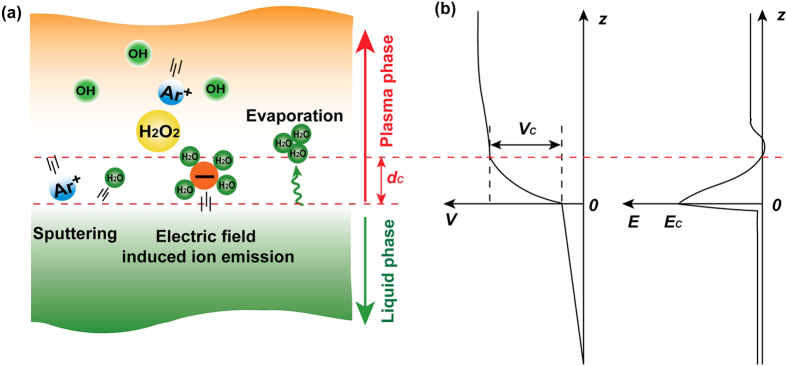
Characteristics of the plasma-liquid interface. **(a)** Three main processes occurring at the plasma-liquid interface as the liquid is cathode, and **(b)** the qualitative characteristics of the voltage potential (*V*) and electric field (*E*) in the plasma-liquid system (ref. 41). *z* is the coordinate along the plasma-liquid direction and at the liquid surface *z* = 0, *V*_*C*_ is the cathode voltage fall, *d*_*C*_ is the thickness of the cathode sheath, and *E*_*C*_ is the electric field at the liquid surface.

Figure 3 depicts three processes contributing to the water transfer from the liquid phase to the gaseous plasma, and the qualitative characteristics of the voltage potential (*V*) and the electric field (*E*) in the plasma-liquid system (ref. 41). Firstly, positive ions in the plasma passing the cathode sheath will be accelerated by the *V*_*C*_, and the constituents of liquid will be sputtered into the gaseous phase by the accelerated energetic ions. This sputtering process has been widely used in low pressure plasma for material fabrication^41^. Secondly, the *V*_*C*_ measured in our case is in the magnitude of ~500 V (see Table 1) which forms an electric field in the order of 100 kV cm^−1^ near the liquid surface (see latter analysis for the electric field estimation). This electric field is high enough to pull out the hydrated negative ions (carrying water molecules) from the liquid surface and transfer them to the gaseous plasma^36^,^42^,^43^,^44^, which is similar to the field emission at a solid surface. Thirdly, there is evaporation at the liquid surface caused by plasma and Joule heating. Obviously, all these three processes can transfer water molecules from the liquid phase into the gaseous plasma, and then the number of water molecules entering the plasma phase is influenced by the above three processes. Water molecules in the gaseous plasma can react with plasma species to form OH, and then H_2_O_2_ is formed by the combination of OH. Finally, H_2_O_2_ dissolves into the liquid to form an aqueous solution. Therefore, H_2_O_2_ production will increase with the number of water molecules entering the plasma phase which is strongly dependent on the above mentioned processes.

The number density of water molecules (*n*) entering the plasma phase can be expressed as


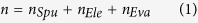


where

*n*_*Spu*_: the number density of water molecules entering the plasma phase due to the sputtering, *n*_*Spu*_ is related to the cathode voltage fall (*V*_*C*_), discharge current (*I*_*d*_), liquid surface tension etc. and it can be described as *n*_*Spu*_ = *k*_*spu*_*I*_*d*_(*E*_*i*_)^1/2^^41^, *k*_*spu*_ is the sputtering coeffieient, and *E*_*i*_ is the energy of incident ion. The positive ion in the plasma obtains its energy by passing the plasma cathode sheath, and therefore the obtained energy is proportional to the *V*_*c*_. Thus, *n*_*Spu*_ can be expressed as *n*_*Spu*_ = *k*_*spu*_*I*_*d*_(*V*_*C*_)^1/2^.

*n*_*Ele*_: the number density of water molecules entering the plasma phase due to the electric field induced hydrated ion emission, and *n*_*Ele*_ is an increasing function of the ion concentration in the liquid phase (*C*_*Ion*_) and the electric field near the liquid surface (*E*_*C*_) but depends on these parameters in a complicated way^44^. We can express it as *n*_*Ele*_ = *f(V*_*C*_, *C*_*Ion*_). In addition, the electric field near the liquid surface (*E*_*C*_) can be estimated as follows. In the cathode sheath, *E* = *E*_*C*_(1 − *z/d*_*C*_), and *V* = *V*_*C*_(*z/d*_*C*_)(2 − *z/d*_*C*_), where *E* is the electric field in the cathode sheath, *z* is the distance away from the liquid surface, *d*_*C*_ is the thickness of cathode sheath, *V* is the voltage potential in the cathode sheath, and *V*_*C*_ is the voltage potential near the liquid surface^41^. If we integrate *E* to get *V*, we can find that *E*_*C*_ = *2V*_*C*_*/d*_*C*_. From the results and analysis in the main paper, we know that the *V*_*C*_ in liquid cathode is in the magnitude of ~500 V, and *d*_*C*_ is taken as 100 μm, and then *E*_*C*_ is estimated to be in the order of 100 kV cm^−1^.

*n*_*Eva*_: the number density of water molecules entering the plasma phase due to evaporation. Based on the thermodynamics law, *n*_*Eva*_ can be expressed as *n*_*Eva*_ = *n*_*0*_ *exp*(*−W/kT*), where *n*_*0*_ is the number density of water molecules in the liquid phase, *W* is the heat of evaporization, *k* is the Boltzmann constant and *T* is the liquid temperature.

Therefore, *n* can be expressed as


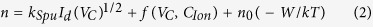


Besides depending on *I*_*d*_ for *n*_*Spu*_, and on *C*_*Ion*_ for *n*_*Eva*_, both *n*_*Spu*_ and *n*_*Ele*_ depend on the cathode voltage fall (*V*_*C*_), and *n*_*Eva*_ depends on the solution temperature.

### H_2_O_2_ energy yields

Based on the discharge voltages shown in Fig. S2, we estimated the average power consumed in the plasma-liquid interactions, and then the H_2_O_2_ generation energy yields were calculated for liquids with different initial conductivities and the results are summarized in Table 2. The H_2_O_2_ energy yields are larger than most of the H_2_O_2_ energy yields generated from discharge plasmas which have been summarized in ref. 28. The maximum energy yield appears for liquid with an initial conductivity of 10500 μS cm^−1^, although the liquid with an initial conductivity of 1.6 μS cm^−1^ produces the largest generation rate. The energy yield can be expressed as


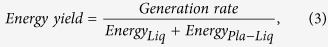


where *Energy*_*Liq*_ and *Energy*_*Pla-Liq*_ are energy consumed in the liquid (mostly taking a form of heating the liquid cylinder) and energy consumed in the plasma-liquid interactions, respectively, and only the latter contributes to the H_2_O_2_ production. Therefore, *Energy*_*Liq*_ can affect the energy yield of the H_2_O_2_ formation. The largest generation rate for the liquid with an initial conductivity of 1.6 μS cm^−1^ can be attributed to the high resistance of the liquid cylinder between the plasma and the graphite electrode. The Joule heating consumes a large portion of the supplied power in the liquid with the initial conductivity of 1.6 μS cm^−1^, but in this case, a larger cathode voltage fall is close to the liquid surface as shown in Table 1, and therefore, it produces a large H_2_O_2_ generation rate with a lower energy yield if compared with the case using liquid with the initial conductivity of 10500 μS cm^−1^. In the plasma-induced H_2_O_2_ generation, to select the liquid with a high or low initial conductivity is dependent on one’s aim: to obtain high energy yield or high generation rate.

## Discussion

Based on the expression of *n*, the water moleclues transfer from the liquid phase to the gaseous plasma is largely determined by the sputtering and the electric field induced hydrated ion emission in the case of liquid cathode since the evaporation for all cases are estimated to be similar [Fig. S6(a)]. The results in Table 1 indicate that the *V*_*C*_ decreases with increasing time for liquids with initial conductivities of 1.60 μS cm^−1^ and 1440 μS cm^−1^, while it almost keeps constant for liquids with initial conductivities of 4800 μS cm^−1^ and 10500 μS cm^−1^. Therefore, from the expression of *n* and the data in Table. 1, one can deduce that *n* for liquids with initial conductivities of 1.60 μS cm^−1^ and 1440 μS cm^−1^ will decrease with increasing time, while it is almost constant for the liquids with initial conductivities of 4800 μS cm^−1^ and 10500 μS cm^−1^. Therefore, the H_2_O_2_ production rate decreases as time increases for liquids with low initial conductivity, while it is almost constant for liquids with high initial conductivity. Because the evaporation for all cases are estimated to be similar [see Fig. S6(a)], *n* is determined by *I*_*d*_*, V*_*C*_*, and C*_*Ion*_. For the case with a constant *I*_*d*_, *n* is related to *V*_*C*_ *and C*_*Ion*_. Although *C*_*Ion*_ is low in case of the liquid with low conductivity (1.6 μS cm^−1^), *n* can be still large since the high *V*_*C*_ (see Table 1) in this case. Therefore, there is a high H_2_O_2_ production rate for the liquid with a low conductivity (1.6 μS cm^−1^). In addition, the estimated *V*_*C*_ are almost the same for discharge currents of 30 mA, 40 mA and 50 mA (~500 V, not shown). When the discharge current increases, the ion flux (related to *I*_*d*_) near the liquid surface is enhanced and thus water transfer from the liquid phase to the gaseous plasma is increased. Consequently, the H_2_O_2_ yield increases with increasing discharge current as shown in Fig. 2(c). The estimated *V*_*C*_ for NaOH solution with an initial conductivity of 4800 μS cm^−1^ is ~30 V higher than that for the NaCl solution with the same conductivity. Based on the above anlysis, one must expect that the NaOH solution should produce more H_2_O_2_. However, the H_2_O_2_ yield for NaOH solution is almost zero as shown in Fig. 2(b). The reason might be as follows. H_2_O_2_ is a weak acid and it can react with OH^−^ to form HO_2_^−^ in concentrated NaOH solution (H_2_O_2_ + OH^−^ → HO_2_^−^ + H_2_O)^45^,^46^,^47^. Therefore, the produced H_2_O_2_ was consumed by reacting with NaOH, resulting in a very low H_2_O_2_ yield.

When liquid acts as anode, the cathode voltage fall is formed on the tungsten steel electrode, and therefore there is no sputtering and electric field induced ion emission at the liquid surface, and evaporation is the only way to transfer water molecules from liquid phase into the gasesous plasma. Compared with the case of liquid cathode, *n* is insignificantly small in the case of liquid anode, resulting in a low H_2_O_2_ yield as shown in Fig. 2 (b). Comprison of OH opitical emission intensity in cases of liquid cathode and anode also confirms this conclusion (Fig. S9).

In summary, using water as the only consumed material, H_2_O_2_ is directly synthesized by plasma-water interations and the H_2_O_2_ production rate can reach 1200 μmol/h when the liquid cathode is purified water or NaCl solution with an initial conductivity of 10500 μS cm^−1^. The H_2_O_2_ production rate strongly depends on the plasma-liquid interactions at the liquid surface: sputtering, high electric field induced hydrated ion emission, and evaporation. Liquid cathode performs much better than liquid anode in producing H_2_O_2_ by plasma-water interations. In addition, the synthesized H_2_O_2_ can be consumed if the liquid contains some constituent able to react with H_2_O_2_ such as NaOH.

## Methods

### Measurement of the H_2_O_2_ yield

Because H_2_O_2_ can react with titanium sulfate. in strong acid to form H_2_TiO_4_ (Ti^4+^ + H_2_O_2_ + 2H_2_O → H_2_TiO_4_ + 4 H^+^) and the absorption intensity of the yellow-coloured H_2_TiO_4_ in 410 nm is proportional to the reacted H_2_O_2_ concentration^15^,^48^,^49^,^50^. We can use it to determine the synthesized H_2_O_2_ concentration. 7.5 ml [Ti(SO_4_)_2_,120 g/l) was added to 250 ml H_2_SO_4_ (1.5 M) to obtain the test solution of titanium sulfate. We used H_2_O_2_ with standard concentrations to obtain the proportionality between the absorption intensity of H_2_O_2_ at 410 nm, and the results are presented in Fig. S8(a). Once the proportionality is obtained, the H_2_O_2_ yield is estimated by the following equation:


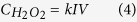


where *k* is the proportionality obtained by linearly fitting Fig. S8(b), *I* is the absorption intensity of synthesized H_2_O_2_ at 410 nm, and *V* is the solution volume (in our case, 400 ml).

### Measurement of the pH value, conductivity, and temperature of the liquid

The pH value and temperature of the solution were measured by a pH detector with a temperature sensor (Yesmylab SX620), and the solution conductivity was measured by a conductivity detector (Yesmylab SX650).

### Electrical characterization of the discharge plasma

The voltage between the tungsten steel and the graphite electrodes was measured by a high voltage (H.V.) probe (Tektronix P6015A) and the current was achieved from dividing the voltage across a 10-Ω resistor which was in series connected with the graphite electrode.

## Additional Information

**How to cite this article**: Liu, J. *et al*. Direct synthesis of hydrogen peroxide from plasma-water interactions. *Sci. Rep.* **6**, 38454; doi: 10.1038/srep38454 (2016).

**Publisher's note:** Springer Nature remains neutral with regard to jurisdictional claims in published maps and institutional affiliations.

## Supplementary Material

Supplementary Information

## Figures and Tables

**Table 1:** Cathode voltage falls (*V*_*C*_) for liquids with different initial conductivities. NaCl was used to adjust the solution conductivity.

Time (min)	*V*_*C*_ (*V*)			
	1.60 μS cm^−1^	1440 μS cm^−1^	4800 μS cm^−1^	10500 μS cm^−1^
0	N/A^[a]^	581	504	512
20	663	559	494	505
40	590	544	507	510
60	558	526	493	506

[a] Data are unavailable because the discharge is unstable in the very beginning for the liquid with an initial conductivity of 1.60 μS cm^−1^.

**Table 2:** Consumed power, energy yield and the generation rate of hydrogen peroxide generation for liquids with different initial conductivities.

Initial conductivities (μS cm^−1^)	Power (10^−3^ kW)	Energy Yield (mg kWh^−1^)	Generation Rate (mg h^−1^)
1.60	38.1	1089	41.48
1440	24.5	866	21.22
4800	23.1	1354	31.28
10500	22.8	1741	39.78

NaCl was used to adjust the solution conductivity.

## References

[b1] Hill C. A Vertical Empire: the History of the UK Rocket and Space Programme, 1950–1971. (World Scientific, 2001).

[b2] Campos‐Martin J. M., Blanco‐Brieva G. & Fierro J. L. Hydrogen peroxide synthesis: an outlook beyond the anthraquinone process. Angew. Chem. Int. Ed. 45, 6962–6984 (2006). 10.1002/anie.200503779 17039551

[b3] Centi G., Perathoner S. & Abate S. in Modern Heterogeneous Oxidation Catalysis: Design, Reactions and Characterization 253–287 (2009).

[b4] Landon P., Collier P. J., Papworth A. J., Kiely C. J. & Hutchings G. J. Direct formation of hydrogen peroxide from H2/O2 using a gold catalyst. Chem. Comm., 2058–2059 (2002). 12357777 10.1039/b205248m

[b5] Nomura Y. . Nanocolloidal Pd‐Au as Catalyst for the Direct Synthesis of Hydrogen Peroxide from H2 and O2. ChemSusChem 1, 619–621 (2008). 18702162 10.1002/cssc.200800029

[b6] Edwards J. K. . The Direct Synthesis of Hydrogen Peroxide Using Platinum‐Promoted Gold–Palladium Catalysts. Angew. Chem. Int. Ed. 53, 2381–2384 (2014). 10.1002/anie.201308067 24474182

[b7] Murayama T. & Yamanaka I. Electrosynthesis of neutral H2O2 solution from O2 and water at a mixed carbon cathode using an exposed solid-polymer-electrolyte electrolysis cell. J. Phys. Chem. C 115, 5792–5799 (2011).

[b8] Yamanaka I., Onizawa T., Takenaka S. & Otsuka K. Direct and Continuous Production of Hydrogen Peroxide with 93% Selectivity Using a Fuel‐Cell System. Angew. Chem. Int. Ed. 42, 3653–3655 (2003). 10.1002/anie.200351343 12916038

[b9] Murayama T. & Yamanaka I. Neutral H2O2 Synthesis by Electrolysis of O2 and Water. ECS Trans. 25, 19–24 (2010). 10.1002/anie.200704431 18224637

[b10] Yamanaka I. & Murayama T. Neutral H2O2 synthesis by electrolysis of water and O2. Angew. Chem. Int. Ed. 47, 1900–1902 (2008). 10.1002/anie.200704431 18224637

[b11] Liu Y., Quan X., Fan X., Wang H. & Chen S. High‐Yield Electrosynthesis of Hydrogen Peroxide from Oxygen Reduction by Hierarchically Porous Carbon. Angew. Chem. Int. Ed. 54, 6837–6841 (2015). 10.1002/anie.201502396 25892325

[b12] Cai R., Hashimoto K., Fujishima A. & Kubota Y. Conversion of photogenerated superoxide anion into hydrogen peroxide in TiO 2 suspension system. J. Elecctroanal. Chem. 326, 345–350 (1992).

[b13] Kormann C., Bahnemann D. W. & Hoffmann M. R. Photocatalytic production of hydrogen peroxides and organic peroxides in aqueous suspensions of titanium dioxide, zinc oxide, and desert sand. Environ. Sci. Technol. 22, 798–806 (1988). 22195664 10.1021/es00172a009

[b14] Cai R., Kubota Y. & Fujishima A. Effect of copper ions on the formation of hydrogen peroxide from photocatalytic titanium dioxide particles. J. Catal. 219, 214–218 (2003).

[b15] Satterfield C. N. & Bonnell A. H. Interferences in titanium sulfate method for hydrogen peroxide. Anal. Chem. 27, 1174–1175 (1955).

[b16] Shiraishi Y. . Sunlight‐Driven Hydrogen Peroxide Production from Water and Molecular Oxygen by Metal‐Free Photocatalysts. Angew. Chem. Int. Ed. 126, 13672–13677 (2014). 10.1002/anie.201407938 25293501

[b17] Venugopalan M. & Jones R. Chemistry of dissociated water vapor and related systems. Chem. Rev. 66, 133–160 (1966).

[b18] Morinaga K. The Reaction of Hydrogen and Oxygen through a Silent Electric Discharge. I. The Formation of Hydrogen Peroxide. Bull. Chem. Soc. Jpn. 35, 345–348 (1962).

[b19] Yi Y. . Safe direct synthesis of high purity H2O2 through a H2/O2 plasma reaction. Angew. Chem. Int. Ed. 52, 8446–8449 (2013). 10.1002/anie.201304134 23804306

[b20] Liu Z. . Physicochemical processes in the indirect interaction between surface air plasma and deionized water. J. Phys. D: Appl. Phys. 48, 495201 (2015).

[b21] Chen Q., Li J. & Li Y. A review of plasma–liquid interactions for nanomaterial synthesis. J. Phys. D: Appl. Phys. 48, 424005 (2015).

[b22] Mariotti D., Patel J., Švrček V. & Maguire P. Plasma–liquid interactions at atmospheric pressure for nanomaterials synthesis and surface engineering. Plasma Process Polym. 9, 1074–1085 (2012).

[b23] Akolkara R. & Sankarana R. M. Charge transfer processes at the interface between plasmas and liquids. J. Vac. Sci. Technol. A 31, 050811 (2013).

[b24] Richmonds C. . Electron-Transfer Reactions at the Plasma–Liquid Interface. J. Am. Chem. Soc. 133, 17582–17585 (2011). 21985430 10.1021/ja207547b

[b25] Rumbach P., Bartels D. M., Sankaran R. M. & Go D. B. The solvation of electrons by an atmospheric-pressure plasma. Nat. Comm. 6, 8248 (2015). 10.1038/ncomms8248 PMC4557361 26088017

[b26] Bruggeman P. & Schram D. C. On OH production in water containing atmospheric pressure plasmas. Plasma Sources Sci. Technol. 19, 045025 (2010).

[b27] Bobkova E., Shikova T., Grinevich V. & Rybkin V. Mechanism of hydrogen peroxide formation in electrolytic-cathode atmospheric-pressure direct-current discharge. High Energy Chem. 46, 56–59 (2012).

[b28] Locke B. R. & Shih K.-Y. Review of the methods to form hydrogen peroxide in electrical discharge plasma with liquid water. Plasma Sources Sci. Technol. 20, 034006 (2011).

[b29] Lukeš P. Water treatment by pulsed streamer corona discharge (2001).

[b30] Burlica R. & Locke B. R. Pulsed plasma gliding-arc discharges with water spray. IEEE Trans. Ind. Appl. 44, 482–489 (2008).

[b31] De Baerdemaeker F., Šimek M., Člupek M., Lukeš P. & Leys C. Hydrogen peroxide production in capillary underwater discharges. Czechoslovak J. Phys. 56, B1132–B1139 (2006).

[b32] Lukes P., Appleton A. T. & Locke B. R. Hydrogen peroxide and ozone formation in hybrid gas-liquid electrical discharge reactors. IEEE Trans. Ind. Appl. 40, 60–67 (2004).

[b33] Lukes P., Dolezalova E., Sisrova I. & Clupek M. Aqueous-phase chemistry and bactericidal effects from an air discharge plasma in contact with water: evidence for the formation of peroxynitrite through a pseudo-second-order post-discharge reaction of H2O2 and HNO2. Plasma Sources Sci. Technol. 23, 015019 (2014).

[b34] Hsieh K. C., Wang H. & Locke B. R. Analysis of Electrical Discharge Plasma in a Gas‐Liquid Flow Reactor Using Optical Emission Spectroscopy and the Formation of Hydrogen Peroxide. Plasma Process Polym (2016).

[b35] Thagard S. M., Takashima K. & Mizuno A. Chemistry of the positive and negative electrical discharges formed in liquid water and above a gas–liquid surface. Plasma Chem. Plasma Process 29, 455–473 (2009).

[b36] Bruggeman P. . Dc excited glow discharges in atmospheric pressure air in pin-to-water electrode systems. J. Phys. D: Appl. Phys. 41, 215201 (2008).

[b37] Chen Q., Kaneko T. & Hatakeyama R. Reductants in gold nanoparticle synthesis using gas–liquid interfacial discharge plasmas. Appl. Phys. Express 5, 086201 (2012).

[b38] Cserfalvi T. & Mezei P. Direct solution analysis by glow discharge: electrolyte-cathode discharge spectrometry. J. Anal. At. Spectrom. 9, 345–349 (1994).

[b39] Chen Q., Kaneko T. & Hatakeyama R. Rapid synthesis of water-soluble gold nanoparticles with control of size and assembly using gas–liquid interfacial discharge plasma. Chem. Phy. Lett. 521, 113–117 (2012).

[b40] Kaneko T., Chen Q., Harada T. & Hatakeyama R. Structural and reactive kinetics in gas–liquid interfacial plasmas. Plasma Sources Sci. Technol. 20, 034014 (2011).

[b41] Lieberman M. A. & Lichtenberg A. J. Principles of Plasma Discharges and Materials Processing. (John Wiley & Sons, 2005).

[b42] Kebarle P. A brief overview of the present status of the mechanisms involved in electrospray mass spectrometry. J. Mass Spectrom. 35, 804–817 (2000). 10934434 10.1002/1096-9888(200007)35:7<804::AID-JMS22>3.0.CO;2-Q

[b43] Thomson B. & Iribarne J. Field induced ion evaporation from liquid surfaces at atmospheric pressure. J. Chem. Phys 71, 4451–4463 (1979).

[b44] Kebarle P. & Tang L. From ions in solution to ions in the gas phase-the mechanism of electrospray mass spectrometry. Anal. Chem. 65, 972A–986A (1993).

[b45] Nicoll W. & Smith A. Stability of dilute alkaline solutions of hydrogen peroxide. Ind. Eng. Chem. 47, 2548–2554 (1955).

[b46] Gurman M., Shcherbak L. & Rasskazov A. Gold and arsenic recovery from calcinates of rebellious pyrite–arsenopyrite concentrates. J. Min. Sci 51, 586–590 (2015).

[b47] Chlistunoff J. & Simonin J.-P. Ionic association of hydroperoxide anion HO2-in the binding mean spherical approximation. Spectroscopic study of hydrogen peroxide in concentrated sodium hydroxide solutions. J. Phys. Chem. A 110, 13868–13876 (2006). 17181346 10.1021/jp065852l

[b48] Alshammari Y. & Hellgardt K. Partial oxidation of n-hexadecane through decomposition of hydrogen peroxide in supercritical water. Chem. Eng. Res. Des 93, 565–575 (2015).

[b49] Dai X. J. . Efficient and Selectable Production of Reactive Species Using a Nanosecond Pulsed Discharge in Gas Bubbles in Liquid. Plasma Process Polym. 306–310 (2015).

[b50] Eisenberg G. Colorimetric Determination of Hydrogen Peroxide. Ind. Eng. Chem. 15, 327–328, doi: 10.1021/i560117a011 (1943).

