# Supplementary material for: Direct synthesis of hydrogen peroxide from plasma-water interactions
Source: Sci Rep. 2016 Dec 5;6:38454. doi: 10.1038/srep38454 (PMC5137161; doi:10.1038/srep38454)
Supplement: Supplementary Information [file srep38454-s1.pdf]

Electronic Supplementary Information for

Direct Synthesis of Hydrogen Peroxide from Plasma-Water Interactions

J. Liu,[1] B. He,[2] Q. Chen,[2,*] J. Li,[3] Q. Xiong,[4] G. Yue,[5] X. Zhang,[2] S. Yang,[2] H. Liu, [2,*] and Q. H. Liu[6]

[1]Institute of Electromagnetics and Acoustics, Department of Materials Science and Engineering, College of Materials, Xiamen University, Xiamen 361005, P. R. China

[2]Fujian Provincial Key Laboratory of Plasma and Magnetic Resonance, Institute of Electromagnetics and Acoustics, Department of Electronic Science, Xiamen University, Xiamen 361005, P. R. China

[3]Key Laboratory of Special Function Materials & Structure Design of the Ministry of Education, Key Laboratory for Magnetism & Magnetic Materials of the Ministry of Education, and School of Physical Science & Technology, Lanzhou University, 222 South Tianshui Road, Lanzhou 730000, China

[4]State Key Laboratory of Power Transmission Equipment & System Security and New Technology, Chongqing University, Chongqing 400044, P. R. China

[5]Department of Materials Science and Engineering, College of Materials, Xiamen University, Xiamen 361005, P. R. China

[6]Department of Electrical and Computer Engineering, Duke University, Durham, NC 27708, USA

E-mail: chenqiang@xmu.edu.cn & liuhai8619@xmu.edu.cn

1. Experimental setup

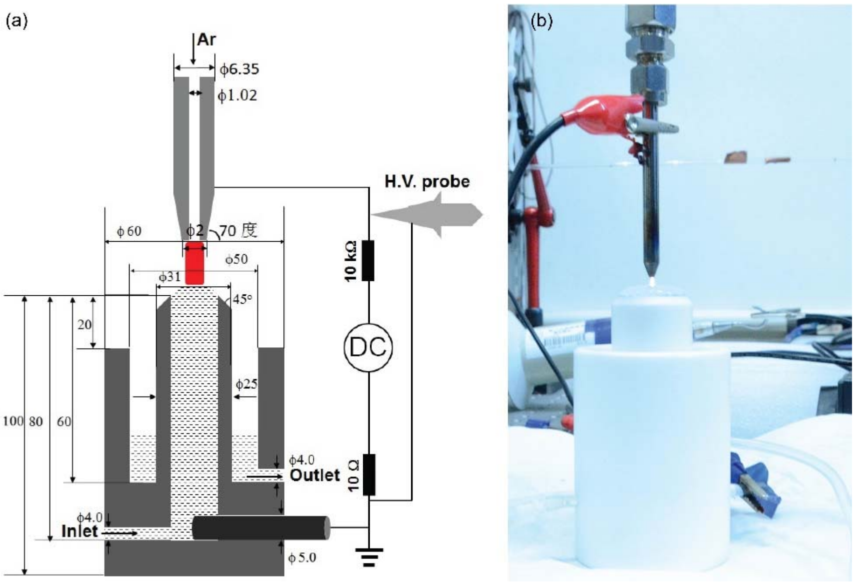

Figure S1. (a) Schematic of the experimental setup (unit: mm), and (b) a photograph of the plasma-liquid interactions.

Figure S1 shows the experimental setup and a photograph of the plasma-liquid interactions. The cylinder-like cell was made from polytetrafluoroethene and its geometry parameters are also indicated in Fig. S1(a). A home-made direct current power source was used to ignite the Ar atmospheric pressure discharge plasma between a tungsten steel tube and an aquesous solution surface. A 10-kΩ resistor was connected in series with the tungsten steel electrode to avoid the plasma transfer from glow-like discharge to arc. The Ar flow rate was 20 sccm. A graphite rod (5 mm in diameter) was placed at the bottom of the solution to act as an inert electrode. The initial conductivity of the solution was adjusted by dissolving NaCl or NaOH in purified water. The purified water has a conductivity of 1.60 μS cm^{-1}. A peristaltic pump was used to circulate the 400 ml solution at a rate of 200 ml/min. Unless stated otherwise, the solution acts as cathode (positive voltage applied to the tungsten steel electrode), the discharge gap was 3 mm, and the discharge current was 30 mA.

The voltage between the tungsten steel and the graphite electrodes was measured by a high voltage (H.V.) probe (Tektronix P6015A) and the current was achieved from dividing the voltage across a 10-Ω resistor which was in series connected with the graphite electrode. The pH value and temperature of the solution were measured by a pH detector with a temperature sensor (Yesmylab SX620), and the solution conductivity was measured by a conductivity detector (Yesmylab SX650).

2. Electrical characterziaiton of the discharge plasma

Figure S2 presents the discharge voltage evolution when the discharge current is 30 mA. The results demonstrate that the voltage for the purified water changes very quickly before 20 min plasma operation, while it almost keeps constant for liquids with high initial conductivity. The variation of the solution conductivity shown in Fig. S3 might account for this phenomenon. Except for NaOH (its conductivity being almost constant), the increase of the solution conductivity is about 600 μS cm^{-1} after 60 min plasma treatment. For the solutions with initial conductivities of 1440 μS cm^{-1} , 4800 μS cm^{-1}, and 10500 μS cm^{-1}, these changes are not enough to change the discharge voltage so much, but for the purified water which has an initial conductivity of 1.60 μS

cm^{-1}, the change of conducivity is relatively large (258 μS cm^{-1} after plamsa treatment of 20 min, almost 160 times of the initial conductivity of 1.60 μS cm^{-1}), and thus the discharge voltage changes very quickly in the first 20 min plamsa treatment in the case of purified water.

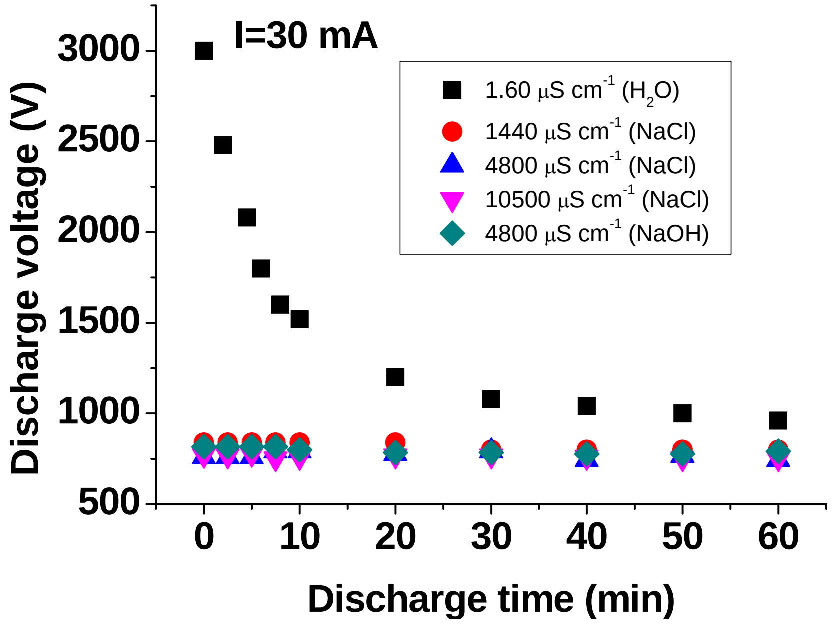

Figure S2. Discharge voltage evolution. The discharge current is 30 mA.

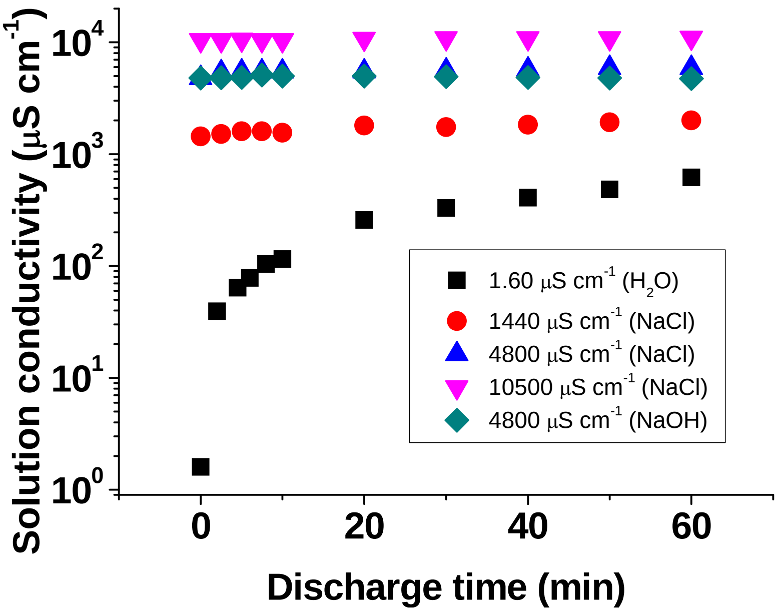

Figure S3. Solution conductivity evolution. The discharge current is 30 mA.

For all the cases, the discharge plasma is a glow-like discharge, and therefore we only show the dependence of the discharge voltage on the discharge current for one case in Fig. S4 which demonstrates a glow-like characteristic of the plasma, i.e., the voltage is almost independent on the current.

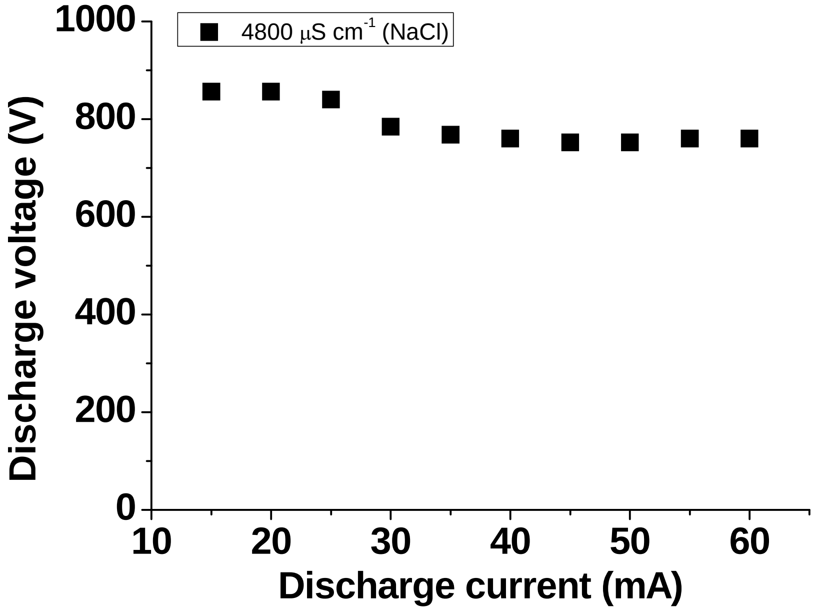

Figure S4. Dependence of the discharge voltage on the discharge current. The gap between tungsten steel tube and the liquid surface is 3 mm and the Ar flow rate is 20 sccm.

3. Investigation of the cathode voltage fall

As shown in Fig. S5, by extrapolating the linear curve of the discharge voltage as a function of the gap between the tungsten steel tube and the liquid surface to zero, we can get an intercept (V_{int}). The cathode voltage fall (V_C) was estimated by $V_C = V_{int} - V_{solution}$, where $V_{solution}$ is the voltage across the graphite electrode and the liquid surface. By considering the resistance ($R_{solution}$) of the liquid cylinder between the graphite electrode and the liquid surface as shown in Fig. 1, $V_{solution}$ can be estimated by $V_{solution} = I_d*R_{solution}$, where I_d is the discharge current, and $R_{solution}$ can be obtained by calculating the liquid cylinder resistance using its conductivity and geometry parameters.

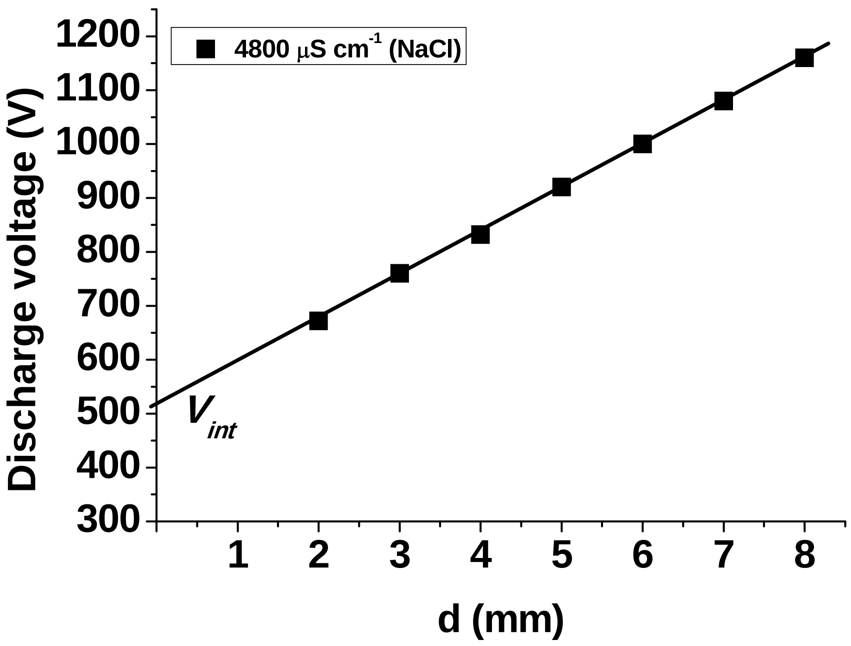

Figure S5. Discharge voltage as a function of the gap between the tungsten steel tube and the liquid surface.

4. The pH value and temperature change of the liquid during plasma treatment

During the plasma treatment, as shown in Fig. S6(a), the solution temperature increases from room temperature (~23 °C) to ~39 °C and the final temperatures for all solutions after 60 min plasma treatment differ within 3 °C. The temperature increase can be accounted to the Joule heating in the bulk solution and plasma induced heating at the solution surface. pH value of the solution decreases with increasing plasma treatment time as shown in Fig. 6(b). Because the plasma-water interactions were performed in an open air atmosphere, N_2 and O_2 can diffuse into the discharge zone and participate in the plasma-water interactions, which leads to the formation of HNO_2 and HNO_3[1-3], and thereby the solution is acidified by the dissolution of HNO_2 and HNO_3.

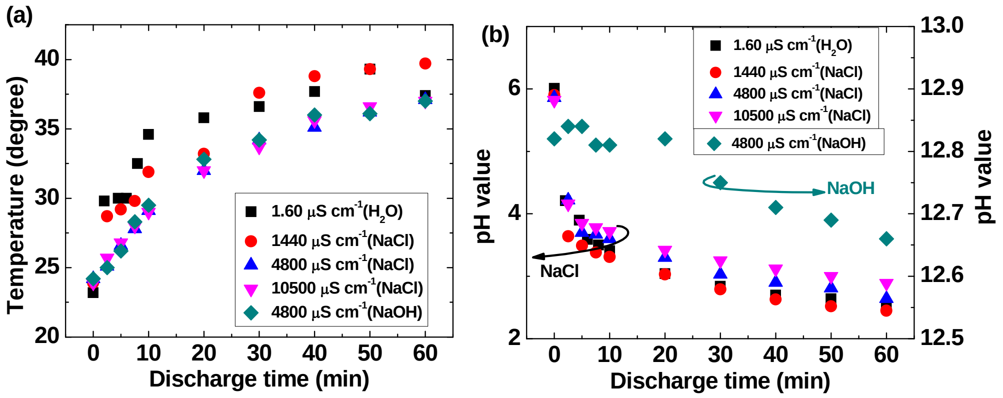

Figure S6. Time evolutions of the liquid (a) temperature, and (b) pH value.

Figure S7 presents a comprison of the time evolution of liquid temperature in both liquid anode and cathode.

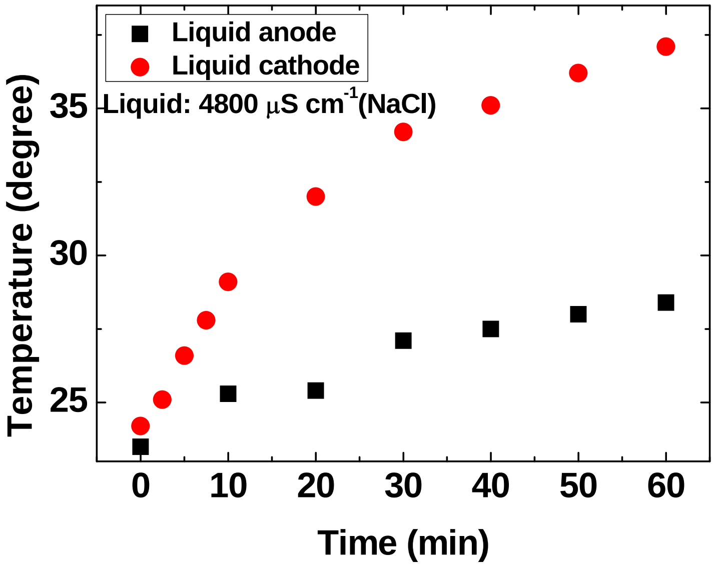

Figure S7. Time evolution of the liquid temperature in both liquid anode and cathode.

5. H_2O_2 yield measurement

Because H_2O_2 can react with titanium sulfate in strong acid to form H_2TiO_4 ($Ti^{4+} + H_2O_2 + 2H_2O \rightarrow H_2TiO_4 + 4H^+$) and the absorption intensity of the yellow-coloured

H_2TiO_4 in 410 nm is proportional to the reacted H_2O_2 concentration[6-9]. We can use it to determine the synthesized H_2O_2 concentration. 7.5 ml [Ti$(SO_4)_2$,120 g/l) was added to 250 ml H_2SO_4 (1.5 M) to obtain the test solution of titanium sulfate. We used H_2O_2 with standard concentrations to obtain the proportionality between the absorption intensity of H_2O_2 at 410 nm, and the results are presented in Fig. S9(a). Once the proportionality is obtained, the H_2O_2 yield is estimated by the following equation:

$$C_{H2O2}=k*I*V \quad (1),$$

where k is the proportionality obtained by linearly fitting Fig. S9(b), I is the absorption intensity of synthesized H_2O_2 at 410 nm, and V is the solution volume (in our case, 400 ml).

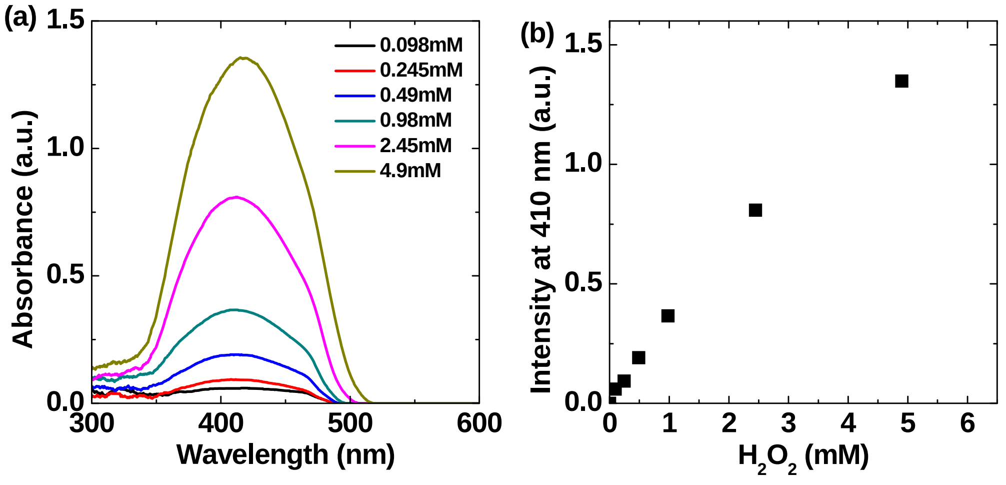

Figure S8. (a) Absorption spectra of H_2O_2 with standard concentrations, and (b) the linear relationship between the absorption intensity at 410 nm and the H_2O_2 concentration.

6. Spectroscopic diagnostic of the discharge plasma

We also compared the optical emission spectra for plasmas using liquid cathode and anode, optical emission spectroscopy (Ocean Optics USB2000+) was used to detect the optical emission spectra and the optical fiber was located near the liquid surface and 20 mm away from the plasma. The results are shown in Fig. S10. Evidently, the emission intensity of OH line (309 nm) in the case with a solution cathode is much

higher than that with a solution anode. Also, Na line (589 nm) is hardly detected in the case of solution andoe, while its emission intensity is very strong in the plasma using a solution cathode. In the case of liquid cathode, Na is drawed out from the solution phase by positive ion sputtering and excited in the discharge zone which finally emits 589 nm light. These results indirectly support our conclusion of the water constituents transfer at the plasma-liquid interface.

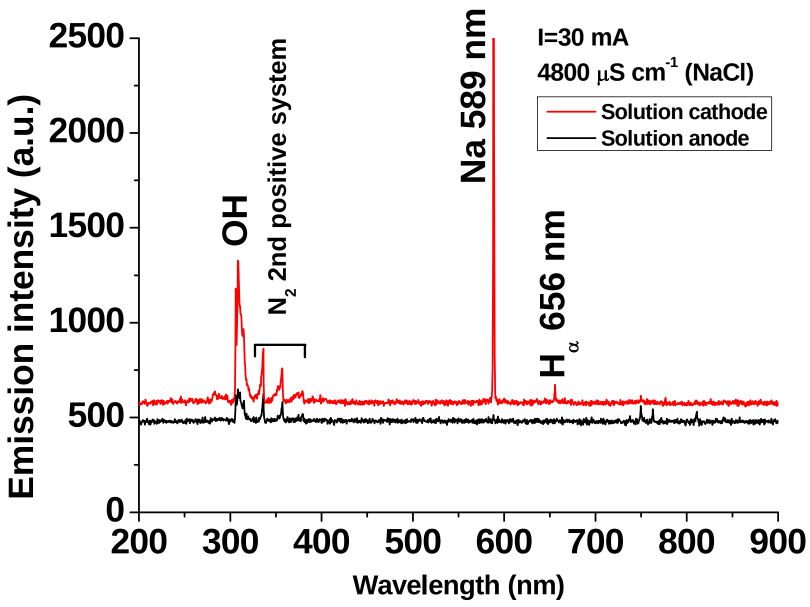

Figure S9. Optical emission spectra of plasmas using a solution cathode and anode.

References

(1) Chen, Q.; Li, J.; Saito, K.; Shirai, H. *J. Phys. D: Appl. Phys.* **2008**, *41*, 175212.

(2) Chen, Q.; Shirai, H. *Eur. Phys. J. D* **2012**, *66*, 1.

(3) Bruggeman, P.; Leys, C. *J. Phys. D: Appl. Phys.* **2009**, *42*, 053001.

(4) Lieberman, M. A.; Lichtenberg, A. J. *Principles of Plasma Discharges and Materials Processing*; John Wiley & Sons, 2005.

(5) Kebarle, P.; Tang, L. *Anal. Chem.* **1993**, *65*, 972A.

(6) Alshammari, Y.; Hellgardt, K. *Chem. Eng. Res. Des* **2015**, *93*, 565.

(7) Satterfield, C. N.; Bonnell, A. H. *Anal. Chem.* **1955**, *27*, 1174.

(8) Dai, X. J.; Corr, C. S.; Ponraj, S. B.; Maniruzzaman, M.; Ambujakshan, A. T.; Chen, Z.; Kviz, L.; Lovett, R.; Rajmohan, G. D.; de Celis, D. R. *Plasma Process Polym.* **2015**.

(9) Eisenberg, G. *Ind. Eng. Chem.* **1943**, *15*, 327.
